# Insights into the clinical management of the syndrome of supine hypertension – orthostatic hypotension (SH-OH): The Irish Longitudinal Study on Ageing (TILDA)

**DOI:** 10.1186/1471-2318-13-73

**Published:** 2013-07-15

**Authors:** Roman Romero-Ortuno, Matthew DL O’Connell, Ciaran Finucane, Christopher Soraghan, Chie Wei Fan, Rose Anne Kenny

**Affiliations:** 1 The Irish Longitudinal Study of Ageing (TILDA), Lincoln Gate, Trinity College Dublin, Dublin 2, Republic of Ireland

**Keywords:** Orthostatic hypotension, Hypertension, Orthostatic intolerance, Syncope, Fainting, Beta-adrenergic blockers, Antidepressive agents, Hypnotics and sedatives, Polypharmacy, Cross-sectional survey

## Abstract

**Background:**

Our previously proposed *morphological* classification of orthostatic hypotension (MOH) is an approach to the definition of three typical orthostatic hemodynamic patterns using non-invasive beat-to-beat monitoring. In particular, the MOH pattern of *large drop/non-recovery* (MOH-3) resembles the *syndrome of supine hypertension–orthostatic hypotension* (SH-OH), which is a treatment challenge for clinicians. The aim of this study was to characterise MOH-3 in the first wave of The Irish Longitudinal Study of Ageing (TILDA), with particular attention to concurrent symptoms of orthostatic intolerance (OI), prescribed medications and association with history of faints and blackouts.

**Methods:**

The study included all TILDA wave 1 participants who had a Finometer® active stand. Automatic data signal checks were carried out to ensure that active stand data were of sufficient quality. Characterisation variables included demographics, cardiovascular and neurological medications (WHO-ATC), and self-reported information on comorbidities and disability. Multivariable statistics consisted of logistic regression models.

**Results:**

Of the 4,467 cases, 1,456 (33%) were assigned to MOH-1 (*small drop, overshoot*), 2,230 (50%) to MOH-2 (*medium drop, slower but full recovery*), and 781 (18%) to MOH-3 (*large drop, non-recovery*). In the logistic regression model to predict MOH-3, statistically significant factors included being on antidepressants (OR = 1.99, 95% CI: 1.50 – 2.64, *P* < 0.001) and beta blockers (OR = 1.60, 95% CI: 1.26 – 2.04, *P* < 0.001). MOH-3 was an independent predictor of OI after full adjustment (OR = 1.47, 95% CI: 1.25 – 1.73, *P* < 0.001), together with being on hypnotics or sedatives (OR = 1.83, 95% CI: 1.31 – 2.54, *P* < 0.001). In addition, OI was an independent predictor of history of falls/blackouts after full adjustment (OR = 1.27, 95% CI: 1.09 – 1.48, *P* = 0.003).

**Conclusions:**

Antidepressants and beta blockers were independently associated with MOH-3, and should be used judiciously in older patients with SH-OH. Hypnotics and sedatives may add to the OI effect of MOH-3. Several trials have demonstrated the benefits of treating older hypertensive patients with cardiovascular medications that were not associated with adverse outcomes in our study. Therefore, the evidence of benefit does not necessarily have to conflict with the evidence of potential harm.

## Background

Orthostatic hypotension (OH) is generally defined as a drop in blood pressure on standing, which in a given subject is regarded as abnormal purely on the basis of its magnitude. As such, OH is a clinical sign and may be symptomatic or asymptomatic [1].

Orthostatic blood pressure changes can be measured by different methods. Most commonly, clinicians use the auscultatory or oscillometric method with sphygmomanometer [2]. As applied to the latter methods, OH is defined by consensus as a sustained reduction of systolic blood pressure (SBP) of at least 20 mmHg or diastolic blood pressure (DBP) of 10 mmHg within 3 minutes of standing [1]. Many clinicians also measure orthostatic hemodynamic changes with non-invasive beat-to-beat finger arterial blood pressure monitors; however, in the latter case the consensus definition of OH may lack clinical relevance [3,4] and there are no internationally agreed cut-offs for the definition of OH.

OH is an independent cardiovascular risk factor and may be practically estimated by systolic reaction only [5]. It is recognised that elevated SBP prior to a standing manoeuvre is directly associated with the magnitude of the SBP drop [6], to the extent that the current consensus definition of OH requires an SBP drop of at least 30 mmHg in patients with supine hypertension [1]. The clinically recognised syndrome of *supine hypertension and orthostatic hypotension* (SH-OH) poses a particular therapeutic dilemma, as treatment of one aspect of the condition may worsen the other [7]. Indeed, in the treatment of combined hypertension and OH in older adults, more questions than answers still remain [8], and little is known on the influences of cardiovascular and neurological medications on this syndrome.

Although most patients with OH are asymptomatic or have few non-specific symptoms [9], a marked orthostatic blood pressure drop may cause symptoms of orthostatic intolerance (OI) such as dizziness, light-headedness, and/or loss or near-loss of consciousness [10,11]. These symptoms are attributed to hypoperfusion of the central nervous system during orthostasis [12]. OI symptoms may correlate with the lowest blood pressure point reached (i.e. nadir), with the magnitude of blood pressure drop (i.e. delta), or with the rate of blood pressure recovery [13,14]. However, OI may also be caused by conditions other than blood pressure changes, such as vestibular [15,16] or psychosomatic [17] disorders. Indeed, OI is a heterogeneous syndrome [18,19].

It has been suggested that postural symptoms (i.e. OI) correlate much more strongly with (pre-)syncope and falls than does OH (i.e. the isolated blood pressure drop sign) *per se*[20,21]. In other words, *if* OH triggers OI symptoms, then syncope is more likely. However, in many real-life situations the latter theoretical sequence is interrupted, as marked OH can be asymptomatic [22,23] and not all instances of OI result in syncope [18,24].

In a previous investigation, a *morphological* classification of OH (MOH) was proposed [25] as an approach to the measurement of three known [26-28] orthostatic hemodynamic patterns using non-invasive beat-to-beat finger arterial blood pressure monitoring. In that study, a gradient of OI was identified across morphological blood pressure patterns: 17.9% in the *small drop/fast over-recovery* (MOH-1), 27.5% in the *medium drop/slow recovery* (MOH-2) and 44.6% in the *large drop/non-recovery* group (MOH-3) (*P* < 0.001). We also showed a gradient of baseline SBP across MOH groups, suggesting that MOH-3 is, in fact, a syndrome of SH-OH. To date, there had been no studies of the SH-OH syndrome using non-invasive beat-to-beat finger arterial blood pressure monitoring.

In view of the above, the aims of the present study were: (1) to replicate the MOH patterns in a large, population-based sample such as the first wave of The Irish Longitudinal Study on Ageing (TILDA, http://www.tcd.ie/tilda), with especial attention to the MOH-3 pattern as a beat-to-beat analogue of SH-OH; (2) to characterise the MOH-3 group with especial attention to associations with cardiovascular and neurological medications, concurrent OI, and history of fainting; (3) to identify predictors of MOH-3 response in the presence of potential confounders; and (4) to assess the effect of MOH-3 towards OI, and the effect of MOH-3 *and* OI towards fainting history, in the presence of confounders. The latter can be seen as a cross-sectional evaluation of the above-mentioned three pathophysiological steps (OH → OI → fainting). A comprehensive investigation of factors associated with each of those three steps has not been conducted to date but would be helpful in order to gain insights into potentially modifiable factors to prevent OH and OI-related faints, particularly in relation to association with prescribed medications in the SH-OH syndrome. The identified factors will then be investigated longitudinally in TILDA.

## Methods

### Setting

The Irish Longitudinal Study on Ageing (TILDA, http://www.tcd.ie/tilda/) is a large prospective cohort study of the social, economic, and health circumstances of community-dwelling older people in Ireland. This study is based on the first wave of data, which was collected between October 2009 and July 2011. The sampling frame is the Irish Geodirectory, a listing of all residential addresses in the Republic of Ireland. A clustered sample of addresses was chosen, and household residents aged 50 and older and their spouses/partners (of any age) were eligible to participate. The household response rate was 62.0%. In the present study, the analytic sample consisted of those aged ≥ 50 from TILDA wave 1.

The study design has previously been described in detail [29,30]. There were three parts to data collection: a computer-assisted personal interview that included detailed questions on sociodemographic characteristics, wealth, health, lifestyle, social support and participation, use of health and social care, and attitudes toward aging; a self-completion questionnaire; and a health assessment that research nurses performed. Health assessments were conducted in a health centre or in the homes of participants; however, only the centre-based assessments included detailed measurements and novel technologies such as beat-to-beat finger arterial blood pressure monitors [31].

### Ethics and consent

Ethical approval was obtained from the Trinity College Dublin Research Ethics Committee, and all participants provided written informed consent.

### Active stand protocol

Subjects underwent a lying-to-standing orthostatic test (active stand) with non-invasive beat-to-beat blood pressure monitoring using digital photoplethysmography (Finometer® MIDI device, Finapres Medical Systems BV, Amsterdam, The Netherlands, http://www.finapres.com).

An appropriate cuff size was applied to the finger as recommended by the manufacturer [32]. Prior to standing, subjects were resting in the supine position for 10 minutes.

The active stand protocol included the use of the automatic *physiocal* function (physiologic calibration that calibrates the finger arterial size at which finger cuff air pressure equals finger arterial blood pressure). We aimed at the beat interval between physiocals being 30 beats or higher before the start of the active stand. Just prior to standing, the *physiocal* was switched off to ascertain a continuous recording during the orthostatic blood pressure changes, and remained switched off until the end of the test.

The height correction unit (HCU) of the Finometer® was zeroed and implemented as per manufacturer’s specifications [32], and was used to compensate for hydrostatic pressure changes on standing. After the ten minutes of supine rest the subjects were asked to stand, unaided, in a timely manner. After standing, systolic, diastolic blood pressure, and heart rate were monitored for three minutes. Throughout the recording subjects stood motionless and in silence with the monitored arm resting extended by the side. Immediately after the test, subjects were asked to report whether they had felt any symptoms of dizziness, light-headedness or unsteadiness (OI: yes or no).

### Active stand data pre-processing

Active stand data analysis required a number of steps including: 1) data quality screening and artefact rejection; 2) pre-processing and filtering; and 3) blood pressure waveform feature extraction. Data were imported and processed in Matlab*®* R2011b. Data records where the HCU had not been properly applied (e.g. sensor fell off during the recording, was zero throughout, contained significant noise, or was inverted due to incorrect placement) were removed from the analysis.

Additional checks were carried out on the data such as ensuring data met the requirement of a minimum length of stand (≥ 90 seconds). A further check examined the total noise in the baseline and stand sections. For this, each record was divided into two sections; baseline pre-stand (baseline) and standing activity (stand) demarked by times before and after the stand. Each of the sections - baseline and stand - was scored in terms of artefact presence separately. The total number of beats within the baseline and stand sections was counted. The proportion of total time containing significant motion artefact as a fraction of the total signal time were used to quantify the amount of noise in the signal using a validated automated algorithm [33,34]. Signals with significant artefact were rejected from the analysis as per pre-defined criteria.

For the final dataset to be used in the analysis, beat-to-beat values were averaged according to the 5-second averages method described by van der Velde *et al*. [35], in order to filter any remaining noise. Following this, features were extracted for each of these records.

### Finometer® features

The following measures were recorded:

● Baseline systolic (SBP), diastolic blood pressure (DPB), and heart rate (HR): defined as the mean value in the time interval −60 seconds to −30 seconds prior to standing.

● SPB, DBP and HR at the lowest blood pressure value after standing (i.e. nadir values, which are generally achieved within 15 seconds after standing [36]).

● SPB, DBP and HR at 30 seconds post-stand.

● SPB, DBP and HR at 60 seconds post-stand.

● SPB, DBP and HR at 90 seconds post-stand.

● SPB, DBP and HR at 110 seconds post-stand.

● Delta (∆SBP, ∆DBP and ∆HR) was defined as the difference between the respective baselines and nadirs.

● We also computed the percentage of SBP recovery (with respect to baseline) by 30 seconds, 60 seconds, and 110 seconds after the stand.

### Characterisation variables

● *Demographics*: age, sex.

● *Orthostatic intolerance* (OI): self-reported symptoms of dizziness, light-headedness or unsteadiness during the active stand (yes or no).

● *Ever had a blackout or fainted*: yes or no.

● *Medications*, based on the WHO Anatomical Therapeutic Chemical (ATC) classification system (http://www.whocc.no/atc_ddd_index/):

*○ Cardiovascular medications*:

▪ C01A: cardiac glycosides.

▪ C01B: antiarrhythmics, class I and III.

▪ C07A: beta blocking agents.

▪ C03: diuretics.

▪ C09A: ACE inhibitors, plain.

▪ C09C: angiotensin II antagonists, plain.

▪ C08C: selective calcium channel blockers with mainly vascular effects.

▪ C08D: selective calcium channel blockers with direct cardiac effects.

▪ C02C: antiadrenergic agents, peripherally acting.

▪ C01D: vasodilators used in cardiac diseases.

▪ C04A: peripheral vasodilators.

*○ Neurological medications*:

▪ N03A: antiepileptics.

▪ N05A: antipsychotics.

▪ N05B: anxiolytics.

▪ N05C: hypnotics and sedatives.

▪ N06A: antidepressants.

*○ Polypharmacy* was defined as the simultaneous use of 5 or more medications.

● *Comorbidities* (self-reported):

○ Hypertension.

*○ Angina*.

*○ Heart attack*.

*○ Heart failure*.

*○ Diabetes*.

*○ Stroke*.

*○ Transient ischaemic attack (TIA)*.

*○ Abnormal heart rhythm*.

*○ Parkinson’s disease*.

*○ Three or more chronic diseases*.

● *Disability* (self-reported): any disability from the list of Independent Activities of Daily Living (IADL).

● *Cognition:* Mini-Mental State Examination (MMSE) score.

### Statistics

All statistical analyses were performed with SPSS (version 18). Descriptives for dichotomous variables were given as percentages (%). Continuous variables were described as mean with standard deviation (SD).

To classify the sample into MOH groups we used, as before [25], an automatic *K-means Cluster Analysis* procedure, which assigns cases to a fixed number of groups (clusters) whose characteristics are not yet known but are based on a set of specified (clustering) variables. We chose three (*k* = 3) as the number of clusters because three were the previously described [26-28] orthostatic hemodynamic patterns. There was no statistical process to arrive at *k* = 3.

The *clustering* variables (i.e. ∆SBP and % of Baseline SBP at 30, 60 and 110 s) were chosen as key *morphological descriptors* of the beat-to-beat orthostatic blood pressure response that we intended to model. The influence of outliers on the *K*-means cluster analysis was minimised by the pre-processing and data cleaning steps as outlined above.

In the *K*-means analysis, the clustering variables were entered unstandardised. It was decided not to standardise the clustering variables as some have argued that standardisation (*z*-scores specifically) can result in misleading conclusions when true group structure is present [37]. The final cluster membership variable was saved to the dataset.

To compare baseline characteristics between those with and without MOH data, we used the t-test or Mann–Whitney U test (as appropriate) for continuous variables, and the Chi-square test for count data.

To test for a linear trend (i.e. gradient) across MOH clusters, we used the Chi-square for trend for dichotomous variables and the Spearman’s rank correlation coefficient for continuous variables.

Multivariable analyses were based on binary logistic regression (forward conditional procedure). This stepwise method of variable selection involves entry testing based on the significance of the score statistic, and removal testing based on the probability of a likelihood-ratio statistic based on conditional parameter estimates. The significance level for entry into the model was set at *P* < 0.05 and for removal was set at *P* < 0.1.

Multicollinearity diagnostics (tolerance and variance inflation factors: VIF) were checked. The multivariable models were repeated in the subsample of those aged ≥70 in order to explore whether the overall findings also applied to those of more advanced age.

For the purpose of the discussion, and given the elevated number of characterisation variables used, we focused on the most statistically significant associations (i.e. *P* < 0.01).

## Results

**Figure 1:**
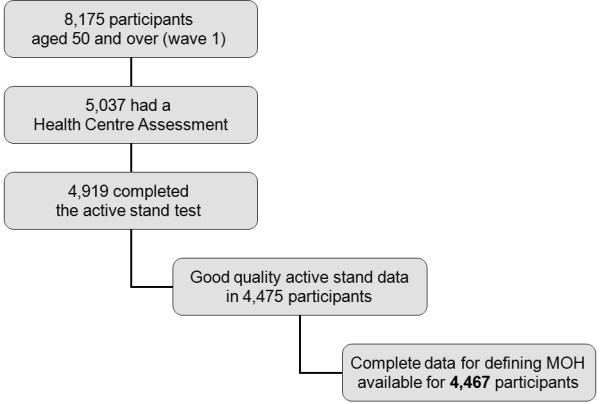
Flowchart of participants.

Of the 8,175 participants aged 50 and over in the first wave of TILDA, 5,037 (62%) had a Health Centre Assessment. Amongst the latter, 4,919 (98%) completed the active stand test. Active stand data were deemed of sufficient quality for analysis in 4,475 participants (91% of active stands). Complete data for defining the MOH groups was available for 4,467 participants. The flowchart of participants is shown in Figure 1.

Table 1 compares characteristics of Health Centre participants with (*N* = 4,467) and without (*N* = 570) MOH data. Those without MOH data were older (mean age 65 *vs*. 62 years, *P* < 0.001), had higher sitting SBP (mean SBP 137 *vs*. 134 mmHg, *P* < 0.001), were taking a higher number of medications (mean 3 *vs*. 2, *P* < 0.001), had higher burden of cardiovascular disease (at least 1: 70% *vs*. 62%, *P* < 0.001) and higher disability burden (at least 1 IADL disability: 10% *vs*. 4%, *P* < 0.001).

**Table 1:** Comparison of Health Centre participants with and without MOH data

	**Has MOH groups (N = 4,467)**	**Missing MOH groups (*N *= 570)**	***P***
Age: mean (SD)	61.6 (8.3)	64.5 (9.4)	**<0.001**^a^
Seated^*^ SBP: mean (SD)	134.3 (19.4)	137.4 (20.0)	**<0.001**^a^
Seated^*^ DBP: mean (SD)	82.3 (11.1)	82.5 (11.5)	0.67^a^
Number of medications: mean (SD)	2.3 (2.5)	2.8 (2.9)	**<0.001**^b^
Any cardiovascular disease: count (%)	2,772 (62.1)	403 (70.1)	**<0.001**^c^
Any IADL disability: count (%)	172 (3.9)	58 (10.1)	**<0.001**^c^

^a^ t-test; ^b^ Mann Whitney U test; ^c^ Chi-square test.

^*^ Seated blood pressure was measured with sphygmomanometer.

**Figure 2:**
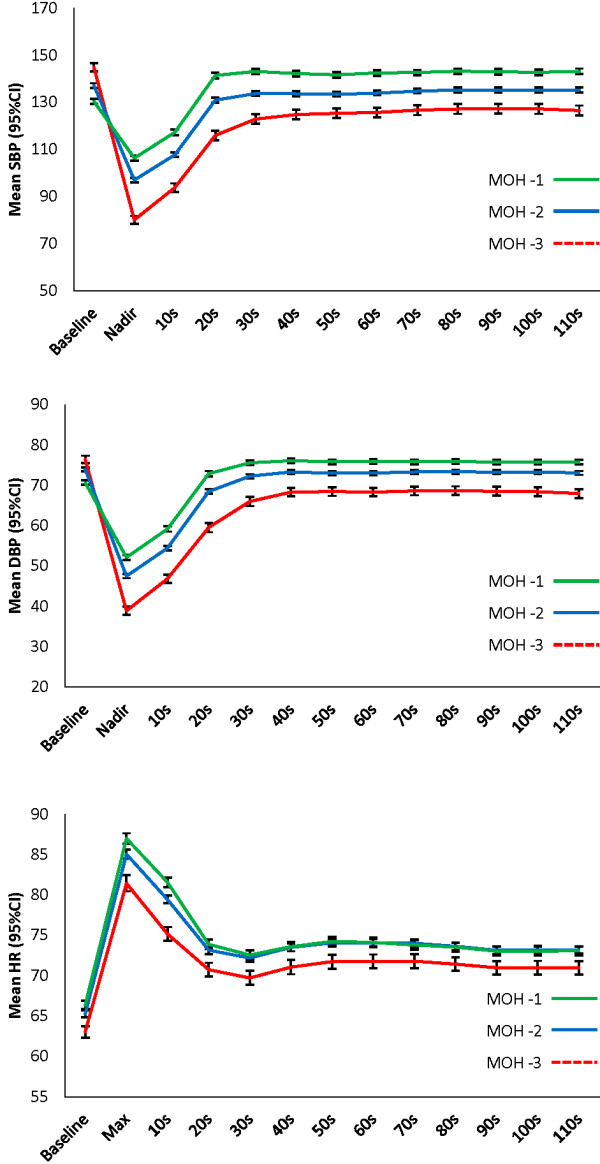
MOH phenotypes (visual description of SBP, DBP and HR behaviour).

In the *K*-means analysis on *N* = 4,467, all clustering variables significantly contributed to the solution (*P* < 0.001). Table 2 shows the characteristics of the three morphological groups and Figure 2 shows their hemodynamic profiles. Of the 4,467 cases assigned to clusters, 1,456 (33%) were assigned to MOH-1 (characterised by a *small drop and overshoot*), 2,230 (50%) to MOH-2 (characterised by a *medium drop and slower but full recovery*), and 781 (18%) to MOH-3 (characterised by a *large drop and incomplete recovery*).

**Table 2:** MOH clusters characterisation

	**MOH-1**	**MOH-2**	**MOH-3**	***P***
	**Small drop, overshoot**	**Medium drop, just recovery**	**Large drop, non-recovery**	
	***N *= 1,456**	***N *= 2,230**	***N *= 781**	
Systolic blood pressure (SBP)				
Baseline SBP (mmHg)	130.4 (20.7)	137.2 (21.3)	144.9 (24.9)	**<0.001**^Σ^
∆ SBP (mmHg)	−24.1 (10.8)	−40.2 (9.9)	−64.8 (15.2)	**<0.001**^Σ^
Nadir SBP (mmHg)	106.3 (22.0)	97.0 (22.1)	80.1 (24.5)	**<0.001**^Σ^
SBP by 30 s (mmHg)	143.1 (22.9)	133.8 (23.0)	122.9 (28.7)	**<0.001**^Σ^
SBP by 30 s (% baseline)	110.0 (8.1)	97.5 (6.6)	84.5 (11.4)	**<0.001**^Σ^
SBP by 60 s (mmHg)	142.5 (23.4)	134.0 (23.2)	125.8 (28.9)	**<0.001**^Σ^
SBP by 60 s (% baseline)	109.5 (8.2)	97.6 (6.6)	86.6 (11.5)	**<0.001**^Σ^
SBP by 90 s (mmHg)	142.9 (23.3)	135.3 (23.0)	127.3 (30.0)	**<0.001**^Σ^
SBP by 90 s (% baseline)	109.9 (8.8)	98.7 (7.1)	87.7 (13.0)	**<0.001**^Σ^
SBP by 110 s (mmHg)	143.2 (23.5)	135.3 (22.8)	126.5 (30.7)	**<0.001**^Σ^
SBP by 110 s (% baseline)	110.1 (9.1)	98.7 (7.1)	87.1 (13.6)	**<0.001**^Σ^
Diastolic blood pressure (DBP)				
Baseline DBP (mmHg)	70.7 (10.4)	74.0 (10.7)	76.4 (13.1)	**<0.001**^Σ^
∆ DBP (mmHg)	−18.5 (7.8)	−26.4 (7.5)	−37.5 (9.7)	**<0.001**^Σ^
Nadir DBP (mmHg)	52.2 (12.3)	47.5 (12.8)	38.9 (14.6)	**<0.001**^Σ^
DBP by 30 s (mmHg)	75.6 (11.6)	72.3 (12.2)	66.0 (16.0)	**<0.001**^Σ^
DBP by 90 s (mmHg)	75.9 (11.3)	73.1 (11.7)	68.3 (15.2)	**<0.001**^Σ^
DBP by 90 s (mmHg)	75.7 (11.0)	73.3 (11.6)	68.6 (15.6)	**<0.001**^Σ^
DBP by 110 s (mmHg)	75.8 (11.3)	73.1 (11.5)	68.0 (15.7)	**<0.001**^Σ^
Heart rate (HR)				
Baseline HR (bpm)	66.4 (10.0)	65.3 (10.0)	63.1 (10.0)	**<0.001**^Σ^
∆ HR (bpm)	20.6 (8.6)	19.8 (8.8)	18.4 (9.5)	**<0.001**^Σ^
Nadir HR (bpm)	87.0 (12.6)	85.1 (13.1)	81.5 (14.2)	**<0.001**^Σ^
HR by 30 s (bpm)	72.6 (11.4)	72.2 (11.8)	69.7 (12.2)	**<0.001**^Σ^
HR by 60 s (bpm)	74.1 (11.4)	74.1 (11.5)	71.8 (12.2)	**<0.001**^Σ^
HR by 90 s (bpm)	73.1 (10.9)	73.1 (11.2)	71.0 (11.9)	**0.001**^Σ^
HR by 110 s (bpm)	73.1 (10.9)	73.2 (11.1)	71.0 (11.9)	**0.001**^Σ^
Orthostatic intolerance and blackouts/faints				
OI symptoms during active stand (%)	33.1	39.4	44.9	**<0.001**^χt^
Ever had a blackout or fainted (%)	17.8	20.2	21.6	0.07^χt^
Demographics				
Age	61.2 (8.1)	61.2 (8.1)	63.6 (9.0)	**<0.001**^Σ^
Age range	50 - 89	50 - 90	50 - 91	**-**
Female gender (%)	48.8	53.4	64.4	**<0.001**^χt^
Medications				
Polypharmacy (5 or more meds) (%)	16.7	16.8	21.0	0.02^χt^
On cardiac glycosides (e.g. digoxin) (C01A) (%)	0.8	0.8	0.5	0.76^χt^
On antiarrhythmics class I and III (C01B) (%)	0.2	0.4	0.4	0.58^χt^
On beta-blocker (C07A) (%)	10.4	10.4	15.7	**<0.001**^**χt**^
On diuretic (C03) (%)	6.2	5.7	6.5	0.66^χt^
On ACE-i (C09A) (%)	10.1	10.9	9.7	0.58^χt^
On ARA (C09C) (%)	7.8	6.8	7.2	0.48^χt^
On calcium channel blocker – with mainly vascular effects (C08C) (%)	7.3	7.3	6.4	0.66^χt^
On calcium channel blocker – with direct cardiac effects (C08D) (%)	1.0	1.3	1.5	0.44^χt^
On peripherally acting anti-adrenergic (e.g. alpha-blocker) (C02C) (%)	0.7	1.8	2.3	**0.004**^**χt**^
On cardiac vasodilator (e.g. nitrates) (C01D) (%)	1.0	1.2	1.7	0.35^χt^
On peripheral vasodilator (C04A) (%)	0.2	0.2	0.3	0.92^χt^
On antiepileptic (N03A) (%)	1.8	2.5	2.4	0.33^χt^
On antipsychotic (N05A) (%)	0.8	0.9	1.8	0.07^χt^
On anxiolytics (N05B) (%)	1.2	1.6	2.7	0.03^χt^
On hypnotics or sedatives (N05C) (%)	3.4	3.3	4.4	0.36^χt^
On antidepressant (N06A) (%)	4.0	5.7	10.2	**<0.001**^**χt**^
Comorbidities				
Hypertension (%)	32.1	32.4	35.3	0.25^χt^
Angina (%)	4.3	3.9	5.9	0.07^χt^
Heart attack (%)	4.3	3.9	3.3	0.51^χt^
Heart failure (%)	0.7	0.9	0.6	0.59^χt^
Diabetes (%)	7.4	6.1	5.9	0.21^χt^
Stroke (%)	1.2	1.1	1.3	0.89^χt^
TIA (%)	1.3	1.7	1.7	0.56^χt^
Abnormal heart rhythm (%)	6.5	7.4	7.3	0.61^χt^
Parkinson’s disease (%)	0.1	0.4	0.5	0.13^χt^
3 or more chronic diseases (%)	23.2	23.7	26.9	0.13^χt^
Disability				
Any IADL disability (%)	3.2	3.8	5.2	0.06^χt^
Cognition				
MMSE score	28.6 (2.0)	28.7 (1.7)	28.6 (1.6)	0.18^Σ^

^Σ^ Spearman’s rank correlation coefficient; ^χt^ Chi-squared test for trend.

### OI and fainting history (Table 2)

Across MOH groups, there was a significant gradient in OI and a non-significant gradient in history of faints/blackouts, in the expected direction (*P* < 0.001 and *P* = 0.065, respectively).

### MOH groups: other gradients (Table 2)

Age-wise, the mean age of participants in MOH-3 was 64 years, while in MOH-1 and MOH-2 it was 61 years. There was an increasing gradient of female sex across MOH clusters (49%, 53% and 64%, respectively).

Twenty-one percent of participants in MOH-3 were on polypharmacy, as opposed to 17% of participants in MOH-1 and MOH-2. Sixteen percent of MOH-3 participants were on beta blockers, compared to 10% in MOH-1 and MOH-2.

Across MOH groups, there was an increasing burden of antidepressants (*P* < 0.001). There was also an increasing burden of peripherally acting antiadrenergic agents (e.g. alpha blockers) (*P* = 0.004) (Table 2).

As regards comorbidity burden, 35% of participants in MOH-3 had history of hypertension, compared to 32% and 32% of MOH-1 and MOH-2 participants, respectively. Twenty-seven percent of MOH-3 participants had three or more chronic diseases, compared to 23% and 24% of MOH-1 and MOH-2 participants, respectively. There were increasing gradients of history of angina and abnormal heart rhythm across MOH clusters, and a decreasing gradient in diabetes. None of these trends reached statistical significance. There was a non significant gradient of increasing IADL disability across clusters (*P* = 0.058) (Table 2).

In the multivariable binary logistic regression model to predict MOH-3 membership (Table 3), the statistically significant factors were: antidepressants (OR = 1.99, 95% CI: 1.50 – 2.64, *P* < 0.001), female sex (OR = 1.73, 95% CI: 1.46 – 2.04, *P* < 0.001), beta blockers (OR = 1.60, 95% CI: 1.26 – 2.04, *P* < 0.001), and age (OR = 1.03, 95% CI: 1.03 – 1.04, *P* < 0.001). In addition, those on peripheral calcium channel blockers were less likely to have a MOH-3 response (OR = 0.68, 95% CI: 0.49 – 0.94, *P* < 0.001). In the subsample of those aged 70 or more, antidepressants, sex, beta blockers, age and peripheral calcium channel blockers still had 95% CIs not including 1 (Table 3).

**Table 3:** Generalised linear model to predict MOH-3 membership

**Full sample**	**B**	**Std. error**	***P***	**Odds ratio**	**95% wald confidence interval for odds ratio**	
					**Lower**	**Upper**
Female sex	0.55	0.08	<0.001	1.73	1.46	2.04
C07A (beta blockers)	0.47	0.12	<0.001	1.60	1.26	2.04
C08C (peripheral CCB)	−0.38	0.16	<0.001	0.68	0.49	0.94
N06A (antidepressants)	0.69	0.14	<0.001	1.99	1.50	2.64
Heart attack	−0.47	0.23	0.042	0.62	0.40	0.98
age	0.03	0.00	<0.001	1.03	1.03	1.04
**Subsample ≥70 years old**	**B**	**Std. error**	***P***	**Odds ratio**	**95% wald confidence interval for odds ratio**	
					**Lower**	**Upper**
Female sex	0.34	0.17	0.040	1.41	1.02	1.96
C07A (beta blockers)	0.44	0.19	0.021	1.55	1.07	2.25
C08C (peripheral CCB)	−0.60	0.27	0.025	0.55	0.33	0.93
N06A (antidepressants)	0.70	0.30	0.018	2.01	1.13	3.60
Diabetes	−0.62	0.33	0.064	0.54	0.28	1.04
Age	0.05	0.02	0.009	1.05	1.01	1.09

Dependent variable: MOH type 3 (large drop, under-recovery). Binary logistic response, forward conditional procedure. Predictors entered: female sex, polypharmacy, C01A (cardiac glycosides), C01B (antiarrhythmics), C07A (beta blockers), C03 (diuretics), C09A (ACE-i), C09C (ARA), C08C (peripheral CCB), C08D (cardiac CCB), C02C (alpha blockers), C01D (cardiac vasodilators), C04A (peripheral vasodilators), N03A (antiepileptics), N05A (antipsychotics), N05B (anxiolytics), N05C (hypnotics, sedatives), N06A (antidepressants), hypertension, angina, heart attack, heart failure, diabetes, stroke¸ TIA, abnormal heart rhythm, Parkinson’s disease, three or more chronic diseases, any IADL disability, age.

In the multivariable binary logistic regression model to predict OI during active stand (Table 4), the statistically significant factors were: hypnotics and sedatives (OR = 1.83, 95% CI: 1.31 – 2.54, *P* < 0.001), MOH-3 (OR = 1.47, 95% CI: 1.25 – 1.73, *P* < 0.001), and history of heart attack (OR = 1.59, 95% CI: 1.16 – 2.19, *P* = 0.004). In addition, advancing age (OR = 0.98, 95% CI: 0.98 – 0.99, *P* < 0.001) and female sex (OR = 0.84, 95% CI: 0.74 – 0.96, *P* = 0.008) were associated with less OI during active stand. In the subsample of those aged 70 or more, being on antiepileptics and having 3 or more chronic diseases seemed to be associated with greater OI, while peripheral calcium channel blockers seemed protective (Table 4).

**Table 4:** Contribution of MOH-3 towards OI in the presence of potential confounders

**Full sample**	**B**	**Std. error**	***P***	**Odds ratio**	**95% wald confidence interval for odds ratio**	
					**Lower**	**Upper**
MOH-3	0.39	0.08	<0.001	1.47	1.25	1.73
Female sex	−0.17	0.06	0.008	0.84	0.74	0.96
N05C (hypnotics, sedatives)	0.60	0.17	<0.001	1.83	1.31	2.54
Heart attack	0.47	0.16	0.004	1.59	1.16	2.19
3 or more chronic diseases	0.18	0.08	0.023	1.19	1.02	1.39
Any IADL disability	0.32	0.16	0.048	1.38	1.00	1.90
Age	−0.02	0.00	<0.001	0.98	0.98	0.99
MMSE	−0.04	0.02	0.043	0.96	0.93	1.00
**Subsample ≥70 years old**	**B**	**Std. error**	***P***	**Odds ratio**	**95% wald confidence interval for odds ratio**	
					**Lower**	**Upper**
C08C (peripheral CCB)	−0.53	0.22	0.015	0.59	0.39	0.90
N03A (antiepileptics)	0.91	0.44	0.036	2.50	1.06	5.86
3 or more chronic diseases	0.38	0.15	0.011	1.46	1.09	1.95

Dependent variable: phasic OI. Binary logistic response, forward conditional procedure. Predictors entered: female sex, polypharmacy, C01A (cardiac glycosides), C01B (antiarrhythmics), C07A (beta blockers), C03 (diuretics), C09A (ACE-i), C09C (ARA), C08C (peripheral CCB), C08D (cardiac CCB), C02C (alpha blockers), C01D (cardiac vasodilators), C04A (peripheral vasodilators), N03A (antiepileptics), N05A (antipsychotics), N05B (anxiolytics), N05C (hypnotics, sedatives), N06A (antidepressants), hypertension, angina, heart attack, heart failure, diabetes, stroke¸ TIA, abnormal heart rhythm, Parkinson’s disease, three or more chronic diseases, any IADL disability, age, MOH-3, MMSE.

In the multivariable binary logistic regression model to predict history of blackouts or faints (Table 5), statistically significant factors were: antiepileptics (OR = 2.39, 95% CI: 1.57 – 3.63, *P* < 0.001), history of abnormal heart rhythm (OR = 1.95, 95% CI: 1.49 – 2.53, *P* < 0.001), history of TIA (OR = 1.93, 95% CI: 1.15 – 3.25, *P* = 0.013), female sex (OR = 1.35, 95% CI: 1.16 – 1.57, *P* < 0.001), antidepressants (OR = 1.62, 95% CI: 1.22 – 2.15, *P* = 0.001), polypharmacy (OR = 1.37, 95% CI: 1.12 – 1.68, *P* = 0.002), and OI during active stand (OR = 1.27, 95% CI: 1.09 – 1.48, *P* = 0.003). In the subsample of those aged 70 or more, antiepileptics, history of abnormal heart rhythm, female sex and polypharmacy were directly associated with history of blackouts or faints.

**Table 5:** Contribution of MOH-3 and OI towards history of blackout or faints in the presence of potential confounders

**Full sample**	**B**	**Std. error**	***P***	**Odds ratio**	**95% wald confidence interval for odds ratio**	
					**Lower**	**Upper**
OI	0.24	0.08	0.003	1.27	1.09	1.48
Female sex	0.30	0.08	<0.001	1.35	1.16	1.57
Polypharmacy	0.32	0.10	0.002	1.37	1.12	1.68
N03A (antiepileptics)	0.87	0.21	<0.001	2.39	1.57	3.63
N06A (antidepressants)	0.48	0.14	0.001	1.62	1.22	2.15
Transient Ischemic Attack	0.66	0.26	0.013	1.93	1.15	3.25
Abnormal heart rhythm	0.67	0.13	<0.001	1.95	1.49	2.53
Age	−0.01	0.00	0.013	0.99	0.98	1.00
**Subsample ≥70 years old**	**B**	**Std. error**	***P***	**Odds ratio**	**95% wald confidence interval for odds ratio**	
					**Lower**	**Upper**
Female sex	0.45	0.19	0.014	1.58	1.10	2.26
Polypharmacy	0.42	0.19	0.028	1.52	1.05	2.20
N03A (antiepileptics)	1.57	0.45	<0.001	4.82	2.00	11.61
Abnormal heart rhythm	0.84	0.24	<0.001	2.31	1.45	3.67

Dependent variable: Ever had a blackout or fainted. Binary logistic response, forward conditional procedure. Predictors entered: female sex, polypharmacy, C01A (cardiac glycosides), C01B (antiarrhythmics), C07A (beta blockers), C03 (diuretics), C09A (ACE-i), C09C (ARA), C08C (peripheral CCB), C08D (cardiac CCB), C02C (alpha blockers), C01D (cardiac vasodilators), C04A (peripheral vasodilators), N03A (antiepileptics), N05A (antipsychotics), N05B (anxiolytics), N05C (hypnotics, sedatives), N06A (antidepressants), hypertension, angina, heart attack, heart failure, diabetes, stroke¸ TIA, abnormal heart rhythm, Parkinson’s disease, three or more chronic diseases, any IADL disability, age, MOH-3, MMSE, OI.

In all multivariable models, all VIF were less than 2, excluding significant multicollinearity.

## Discussion

In the present study, we replicated and characterised the MOH groups and assessed the association of MOH-3 (postulated as a beat-to-beat equivalent of the SH-OH syndrome) on concurrent OI and fainting history. The advantage of the present study is that it is based on a large population-based sample, as opposed to the previous study [25], which was based on a small convenience sample. The aim was to identify potentially modifiable factors to prevent OH and OI-related faints, particularly in relation to association with prescribed medications, and inform further longitudinal studies in TILDA. Naturally, in view of the observational and cross-sectional nature of the study, results need to be interpreted with caution and represent ‘insights’ rather than confirmed signals.

Results highlighted the association of MOH-3 with non-modifiable risk factors such as age and sex, even in the subsample of those aged 70 or more. Consistent with previous literature, advancing age is associated with hypertension [38] and greater orthostatic blood pressure drops [39,40]. There are also known differences in postural autonomic modulation between men and women, which might make women less able to compensate for drops in blood pressure in response to positional changes [41].

In terms of potentially modifiable factors, our findings highlight the important influence of certain types of medications (particularly *antidepressants* and *beta blockers*) in contributing to an SH-OH response (even in those aged ≥70), which may potentially lead to clinically adverse consequences (e.g. complaints of OI). In particular, results strengthen the hypothesis on the relationship between OH and antidepressant pharmacotherapy [42], and also between depressive symptoms and impaired orthostatic blood pressure response. It is known that OH and OI are more frequent in depressed older adults [43], and recent studies have found evidence for an association between the degree of orthostatic SBP drop and brain white matter hyperintensities volume in late-life depression [44,45].

Our results also highlight the association between beta blockers and an impaired orthostatic blood pressure response. The higher burden of beta blockers in MOH-3 subjects is consistent with their lower baseline heart rate and poorer orthostatic heart rate response (Table 2). Beta receptors have been implicated in the pathophysiology of OH [46]; indeed, a known primary autonomically mediated mechanism for maintenance of mean arterial pressure and orthostatic tolerance in healthy subjects is beta adrenergic-induced tachycardia [47]. Our results agree with previous observations that the pressor effects of beta blockers on standing blood pressure may be harmful for older patients with OH [48].

An interesting insight is the potential protective effect of peripheral calcium channel blocker (CCB) medications against MOH-3 (also present in the older subgroup), which is supported by the literature. For example, in a previous study, the peripheral CCB Nilvadipine did not aggravate OH in a sample of patients with Alzheimer’s disease, despite significant reduction in the SBP of treated patients [49]. In another study in hypertensive patients, the peripheral CCB Cilnidipine showed significant decreases in blood pressure without adverse OH effects [50]. Furthermore, a study comparing the influences of anti-anginal drugs on cardiovascular responsiveness to orthostasis found that a dihydropyridine CCB influenced the latter less than a mononitrate or a beta-blocker [51].

On bivariate analyses, we found an increasing burden of peripherally acting antiadrenergic agents (e.g. alpha blockers) across MOH groups. It is likely that the number of patients on alpha blockers was too small to detect an independent effect in multivariable analyses. Yet, the alpha(1)-adrenergic receptor pathway is known to be critical in the recovery from initial OH to prevent cerebral hypoperfusion and ultimately syncope [52], so the clinical relevance of alpha blockers in the SH-OH syndrome might be significant.

Our results support previous findings that OI is a wider, more complex syndrome than the mere sign of having an impaired orthostatic hemodynamic response [19]. In the full sample, MOH-3 was as an independent predictor of OI, but the latter was not the case in the older subsample. In terms of non-modifiable risk factors, and in the full sample, OI was less likely to be reported by females and at advancing age. Again, these age and sex effects merit further investigation but, since they are not modifiable, were not the focus of our study. Previous studies have focused on sex-related differences in OI [53,54]. As regards the age effect, a previous study investigating the changing face of orthostatic and neurocardiogenic syncope with age found that symptomatic patients were significantly younger than asymptomatic [55], which is in keeping with our results.

Although the age and sex effects are non-modifiable, they could be relevant in terms of the postulated sequence of pathophysiological steps (OH → OI → fainting). In short, female and older participants were more likely to have an MOH-3 pattern (Table 3); however, when OI was the dependent variable and MOH-3 membership a covariate, being female and older were significantly less likely to have OI (Table 4). And when MOH-3 and OI were included in a model with blackouts or faints as the dependent variable, being female was once again significantly associated with increased risk (Table 5). The differential effects of age and sex are difficult to explain in our cross-sectional design. However, in terms of the age effect, it is plausible that advancing age may lead to an impairment of *both* the orthostatic hemodynamic response (i.e. more MOH-3) *and* the awareness of the latter (i.e. less OI). Indeed, we know that awareness of orthostatic hypotension is influenced by age: in younger subjects it is usually brief but symptomatic whereas in older individuals the situation is reversed [56,57].

In terms of the differential effects of sex in the pathophysiological sequence OH → OI → fainting, it is difficult to explain why women had more MOH-3, more history of faints, but less OI. OI is much more common in young women relative to men, children or older women [58,59], and women in our sample were middle-aged and older. Another possibility is the presence of sex differences in the self-report of OI; for example, a previous study showed that symptoms of vertigo, dizziness or unsteadiness may be more related to psychological factors in men [60]. The full understanding of this sex effects requires purpose-designed research.

According to our full sample results, OI is more likely to be reported by more co-morbid and disabled patients with MOH-3 hemodynamic pattern, on whom it would be prudent to avoid the use of hypnotics and sedatives. Indeed, hypnotics and sedatives may add to the OI effect of MOH-3, and this is consistent with previous observations on the reduced tolerability of benzodiazepines in the elderly [61] and the greater incidence of OI in older subjects taking sedatives and hypnotics [62]. In keeping with the latter, and consistently in meta-analyses and systematic reviews, the use of sedatives and hypnotics, antidepressants, and benzodiazepines has been shown to be significantly associated with falls in older individuals [63,64].

People with history of faints or blackouts may suffer from conditions not directly related to OH such as epilepsy or cardiac syncope (hence the association with anti-epileptics and anti-arrhythmics). However, in both full and older samples, polypharmacy (i.e. being on five or more regular medications) was independently associated with history of blackouts or faints. We know that age-related physiologic impairments of heart rate, blood pressure, baroreflex sensitivity, and cerebral blood flow, in combination with a higher prevalence of comorbid disorders and concomitant medications (including polypharmacy), account for the increased susceptibility of older persons to syncope [65].

In the full sample, phasic OI was more common in those with history of faints or blackouts, suggesting that part of the latter syndrome may be of hemodynamic nature [66]. Interestingly, faints or blackouts were not significantly related to MOH-3, which supports previous opinion that postural symptoms (i.e. phasic OI) correlate much more strongly with endpoint clinical events such as (pre-)syncope, blackouts and recurrent unexplained falls than does OH (i.e. the isolated blood pressure drop sign) *per se*[20,21]. Indeed, it would appear that the above-mentioned three pathophysiological steps (OH → OI → fainting) operate as a chain, so the prevention of endpoint clinical events could be tackled both at improving OH hemodynamics and at minimising OI as a *mediator*[21]. We know that fall preventive interventions should be provided to older people by a structured, multifaceted approach [67].

As in the previous pilot MOH investigation [25], there was an increasing gradient in baseline SBP across clusters, but only MOH-3 had a mean baseline SBP in the hypertension range (i.e. ≥ 140 mmHg). Indeed, MOH-3 resembles the *syndrome of supine hypertension–orthostatic hypotension* (SH-OH), as applied to beat-to-beat orthostatic blood pressure data. We agree with previous recommendations that, of all patients with SH‒OH, those who have OI require the most clinical attention [7]. In patients with SH-OH, the avoidance of medications that may exacerbate OH and OI and the judicious use of antihypertensive classes that are less likely to aggravate postural blood pressure changes may be safe and adequate approaches to the treatment of this challenging condition [68].

Interestingly, antihypertensive types that have shown consistent benefits in the treatment of hypertension in the very elderly (e.g. ACE-i and diuretics, as in the HYVET trial [69,70]) were not linked with any of our adverse outcomes (i.e. MOH-3, OI, history of faints). Several other studies (e.g. SYST-EUR, CONVINCE, VALUE) have demonstrated the benefits of treating aged hypertensive patients with cardiovascular medications that were not associated with adverse outcomes (e.g. angiotensin receptor antagonists), or seemed even protective (i.e. peripheral CCB) in our study [71]. A Cochrane systematic review established that treating healthy older persons with hypertension is highly efficacious, and that benefits of treatment with low dose diuretics or beta-blockers were clear for persons in their 60s to 70s with either diastolic or systolic hypertension [72]. However, this Cochrane review concluded that differential treatment effects based on patient risk factors, pre-existing cardiovascular disease and competing co-morbidities could not be established from the published trial data [72]. Our study sheds light into the latter limitation and supports the overall conclusion that, in treating older and frailer hypertensive patients, the evidence of benefit does not necessarily have to conflict with the evidence of potential harm.

A number of limitations in this study must be noted. Firstly, its observational cross-sectional design precludes the inference of causality relationships and direct extrapolation of associations. As we stated above, results are to be interpreted with caution and represent ‘insights’ rather than confirmed signals.

Secondly, despite the large total sample size, we know that the 38% of participants who did not have a Health Centre assessment were more likely to have lower socio-economic status, higher levels of physical disability, and were weaker (handgrip strength) and slower (walking speed) than Health Centre respondents [31]. In addition, as Table 1 showed, participants who attended the Health Centre assessment but had no MOH data were older, more hypertensive, more medicated, and more comorbid and disabled than those whose active stand data were included. For these reasons, the frailest in the population may have been underrepresented in the analytic sample. Precise information as to what made participants not complete each stage of the participants’ flow chart (Figure 1) is not available, but frailty-related reasons are very likely. It is known that frailty is associated with missing data in research designs that involve the collection of physical performance measures (i.e. as required in the active stand) [73].

The statistical techniques employed in our analyses also have limitations. Indeed, the *K*-means cluster analysis is exploratory in nature, and the scale and variability of the clustering variables may affect results in unstandardised analyses [74]. However, we replicated here the exact same *K*-means clustering method as previously conducted in a different, smaller and convenient sample [25], leading to very similar results in terms of the characterisation of the MOH clusters. Hence, it is plausible that these three MOH groups exist ‘out there’ in clinical practice, although naturally we cannot confirm their existence as a true group structure. There is ongoing work in TILDA in this area.

As stated above, some of the results from the logistic regression models should be interpreted with caution due to the nature of how the multivariate logistic models were developed, and especially due to the presence of a large number of covariates in the models. In addition, only few participants were on medications potentially associated to MOH-3 such as peripheral vasodilators or antiarrhythmics, so results for the latter classes may have been underpowered.

A limitation of the active stand protocol is that information was lacking on precise dosages, time of ingestion of, and compliance with, the reported medications. Even though polypharmacy was used as a control variable in all models, specific drug interactions towards the outcomes of interest could not be investigated. Limitations of the active stand test itself include its known diurnal variability and relationship with meals [1].

Finally, a limitation of the present study is the impact of *frailty* on orthostatic hemodynamic responses, OI and faints/blackouts. Given that the latter are complex disorders involving multiple physiological systems, the inclusion of frailty may have complemented the inclusion of comorbidity, disability and cognition in the models. For example, older adults without measured hypertension, who are not on an anti-hypertensive medication, appear to have high physiological reserve in general [75]. Unsurprisingly then, many of the people with OH have other many health deficits as well, which can combine to make the person frail and when frailty is taken into account, the specific influence of OH on risk is greatly attenuated, even becoming no longer statistically significant [76]. This is an important area of ongoing work in the longitudinal dimension of TILDA.

## Conclusions

In the present study, we replicated our previously proposed *morphological* classification of orthostatic hypotension (MOH, intended for beat-to-beat monitoring) in the first wave of The Irish Longitudinal Study on Ageing, and we found that the clinical associations were similar as previously reported (e.g. association with OI) [25]. In addition, we proposed the MOH-3 pattern as a beat-to-beat analogue of SH-OH and studied its associations with cardiovascular and neurological medications, concurrent OI, and history of fainting.

Our findings offer cross-sectional insights that, if further validated, may inform the development of clinical guidelines for the treatment of SH-OH. Based on the results of the current study, in a typical clinical setting using phasic orthostatic blood pressure measurements, MOH-3 should be recognised by the presence of baseline hypertension (>140 mmHg), an initial orthostatic blood pressure drop greater than 40 mmHg, and failure to recover 90% of the baseline blood pressure after 2 minutes of standing. If a patient fulfilling those criteria has complaints of OI, then his/her risk of (pre-)syncope, falls and blackouts is higher and clinicians should avoid (if possible) medications that may exacerbate OH and OI (such as beta blockers, antidepressants and hypnotics and sedatives), and make a judicious use of antihypertensives that are less likely to aggravate postural blood pressure changes. Naturally, this should be done within a wider multifaceted approach.

## Competing interests

The authors declared that they have no competing interest.

## Authors’ contributions

All authors: 1) made substantial contributions to conception and design, or acquisition of data, or analysis and interpretation of data; 2) were involved in drafting the manuscript or revising it critically for important intellectual content; and 3) gave final approval of the version to be published.

## Pre-publication history

The pre-publication history for this paper can be accessed here:

http://www.biomedcentral.com/1471-2318/13/73/prepub
